# Which Subgroup of First-Episode Schizophrenia Patients Can Remit During the First Year of Antipsychotic Treatment?

**DOI:** 10.3389/fpsyt.2020.00566

**Published:** 2020-06-19

**Authors:** Zhang Cheng, Yanbo Yuan, Xue Han, Lei Yang, Xin Zeng, Fude Yang, Zheng Lu, Chuanyue Wang, Hong Deng, Jingping Zhao, Xin Yu

**Affiliations:** ^1^Peking University Sixth Hospital, Peking University Institute of Mental Health, NHC Key Laboratory of Mental Health (Peking University), National Clinical Research Center for Mental Disorders (Peking University Sixth Hospital), Beijing, China; ^2^Peking University Clinical Research Institute, Beijing, China; ^3^Beijing Hui-Long-Guan Hospital, Beijing, China; ^4^Tongji Hospital of Tongji University, Shanghai, China; ^5^Beijing Anding Hospital, Capital Medical University, Beijing, China; ^6^West China Hospital, Sichuan University, Chengdu, China; ^7^Mental Health Institute, Second Xiangya Hospital, Central South University, Changsha, China

**Keywords:** first-episode, schizophrenia, antipsychotic, remission, negative symptom

## Abstract

**Objective:**

To investigate the persistent remission rate (PRR) and its predictors within the first year of antipsychotic treatment in first-episode schizophrenia (FES) patients.

**Methods:**

In a sample of 301 FES patients who remained in antipsychotic treatment for 1 year, we assessed symptoms with the Positive and Negative Syndrome Scale (PANSS), cognition in six domains and functioning with the Personal and Social Performance Scale (PSP).

**Results:**

In total, 75.4% (227/301) of FES patients remaining in antipsychotic treatment reached persistent remission (PR) in one year. The PSP score was higher in remitters than non-remitters at the endpoint of the 1-year follow-up (P <0.0001). The PANSS negative score—but not the PANSS total score, positive score or general psychopathological score; PSP score; or cognitive performance at baseline—was negatively associated with PR. Lower scores for “abstract thinking” and “stereotyped thinking” were independent predictors of PR.

**Conclusions:**

In FES, nearly 3/4 patients could achieve PR with 1 year of antipsychotic treatment, and having fewer negative symptoms, especially thinking and volition symptoms, can predict PR.

**Clinical Trial Registration:**

www.ClinicalTrials.gov, identifier NCT01057849.

## Introduction

The prognosis of schizophrenia is considered much worse than that of other psychoses (1). Patients with a first episode of schizophrenia tend to respond better to antipsychotics than chronic patients (2). Various guidelines suggest that first-episode schizophrenia (FES) patients should remain on antipsychotics for at least 1 year for maintenance treatment (3). However, only some FES patients achieve remission in one year of antipsychotic treatment, with remission being the basic aim of such treatment and the basis for an ideal outcome.

It has been reported that the remission rate in FES patients is between 17.0 and 78.0% (4), and this wide range is partly due to the diverse criteria for remission. Remission defined only based upon symptom severity is not stable; approximately 70% of symptom remitters in FES experience symptom exacerbation within 1 year (5). In 2005, Andreasen et al. (with the Remission in Schizophrenia Working Group, RSWG) proposed criteria for persistent remission (PR) in schizophrenia: when eight items on the Positive and Negative Symptoms Scale (PANSS) received a score of less than four points (which was considered to indicate symptomatic remission) for more than 6 months. These criteria were considered to provide researchers and clinicians with a robust, well-defined outcome goal for the long-term treatment of schizophrenia and to facilitate comparison between different studies (6). Jager et al. revealed that the consistency between the criteria by the RSWG (including both symptom severity and minimum time threshold) and other criteria employed previously was between 52.6 and 80.0% (7).

Most studies reporting the persistent remission rate (PRR) of FES patients throughout 1 year of antipsychotic treatment were randomized controlled trials (RCTs) (8, 9). However, due to the strictness of the study design, patients in RCTs are assigned a certain antipsychotic and are excluded from the analysis if they switch to another antipsychotic. Approximately one-third of FES patients require a change in medication within their first year of treatment (10). Thus, the remission rate reported in traditional RCTs may reflect remission from one specific antipsychotic rather than a real-world picture of PRR throughout the 1-year antipsychotic treatment.

Most non-RCT longitudinal studies reported remission rates with criteria other than the criteria by the RSWG (11–15). Lally et al. conducted a meta-analysis of first-episode psychosis (FEP) studies that included longitudinal studies and excluded RCTs (16). According to the results, the symptomatic remission rate in FES reached 56.0%, as judged by diverse criteria and based on a wide follow-up duration ranging from 1 to 17 years. Only two of the included studies reported a PRR based on the criteria by the RSWG. One was conducted in Indonesia and followed 59 FES patients for 17 years. It was reported that 23.7% of the patients achieved both symptom and functional remission, and the remitters ceased antipsychotic use beginning in the 11th year (17). The other study was from Hong Kong and reported a PRR of 59.6% within 1 year in a sample of 104 FES spectrum disorder patients; however, medication information was not reported.

Thus, the PRR of FES during 1 year of antipsychotic treatment remains unclear. We aimed to examine the PRR and its predictors during 1 year of antipsychotic treatment for FES. We hypothesized that 1) more than half of the FES patients could remit during the 1-year antipsychotic treatment and 2) baseline sociodemographic, clinical and cognitive characteristics would predict PR.

## Methods

### Participants and Setting

The data came from a 1-year randomized, open-label clinical trial conducted in 6 major psychiatric hospitals in China from 2008 to 2010 (18). The trial was approved by the ethics committees of the participating centers. Written informed consent was obtained after the participants received a complete description of the study.

FES patients with the following characteristics were recruited: illness duration of fewer than 3 years (the onset was determined in the SCID-I/P according to when the first symptom appeared), continuous antipsychotic treatment for fewer than 4 weeks and a cumulative period of intermittent antipsychotic treatment of fewer than 12 weeks. A 3-phase design was employed to mimic the reality of clinical practice (18). Phase I was a randomization phase (comparison of the three antipsychotics (risperidone, olanzapine and aripiprazole) has been published) (19). Patients who showed little or no benefit from the Phase 1 treatment were allowed to enter Phase 2, in which patients underwent a medication switch to one of the other two antipsychotics. Patients who did not respond to the second antipsychotic were allowed to enter Phase 3, which allowed the research psychiatrists to introduce other antipsychotics such as clozapine and augmentation or antipsychotic polypharmacy. Concomitant treatments such as antidepressants or mood stabilizers were permitted if necessary. The design aimed to keep the patients on antipsychotic treatment for as long as possible. The primary outcome in the original RCT was the baseline-to-endpoint change in the PANSS total score (19). This study was a secondary analysis of an RCT database and based on data of subjects who completed the 1-year follow-up. In total, 569 patients were recruited, and 301 subjects completed the 1-year follow-up (20). Data from the 301 patients were included in the analysis. There were no differences in the demographic, psychopathological and functional data between those who completed the 1-year follow-up and those who did not (P >0.05).

### Assessment and Evaluation

Basic demographic data were collected with a standard data collection form designed especially for this study. Clinical characteristics, including illness duration, acute onset (less than 3 months from symptom onset to full disease manifestation), and early onset (onset before 18 years) was drawn from the SCID-I/P. Psychopathology was measured with the PANSS (21). Function was evaluated with the Personal and Social Performance Scale (PSP) (22). Recruited patients were evaluated by a trained psychiatrist in each hospital at baseline and at week 4, 8, 12, 26, 39 and 52. Six cognitive domains (verbal learning and memory with the Hopkins Verbal Learning Test-Revised [HVLT-R]; visual learning and memory with the Brief Visuospatial Memory Test-Revised [BVMT-R]; speed of processing with the Trail Making Test Part A, Color Trails Test, category fluency test, Stroop Color Test and Wechsler Adult Intelligence Scale digit symbol-coding subtest; working memory and attention with the Wechsler Memory Scale-spatial span subtest and paced auditory serial addition task; executive function with the Stroop Color-Word Test and Color Trails Test; and motor skills with the Grooved Pegboard Test) were measured at baseline (23, 24).

There were two to three psychiatrists responsible for the clinical assessments at each site. All interviewers attended a 1-week training workshop on the use of the rating instruments (the PANSS, PSP and cognitive tests) prior to the study and reached a high level of consistency (intraclass correlation coefficients or kappa values >0.75).

### Primary Outcome

The primary outcome was PRR within 52 weeks, which was calculated as the number of patients who achieved PR within 52 weeks divided by the number of patients who completed the 52-week follow-up. PR was defined with the PANSS as a score on items P1, P2, P3, N1, N4, N6, G5, and G9 ≤3 for at least 6 consecutive months (6). The secondary outcomes were predictors of PR within 52 weeks.

### Statistical Methods

Independent T-tests or nonparametric tests were used to compare the demographic, psychopathological and functional data between remitters and non-remitters. A chi-square test was used to compare categorical data, including gender and originally assigned antipsychotic.

Any variable that was significant in the univariate analysis was confirmed in the multivariate logistic regression analysis with the scores of the six cognitive domains (reported as diathesis factors in schizophrenia).

A final multivariate logistic analysis was conducted on the variables that were confirmed to be significant in the multivariate logistic analysis mentioned above to explore potential interactions.

## Results

### Rate of Remission Within 1 Year of Antipsychotic Treatment

At the endpoint of the 1-year follow-up, 75.4% (227/301) of patients had achieved PR. In total, with the early discontinuers included in denominator, 39.9% (227/569) of patients achieved PR. At the endpoint, the PSP score was higher in remitters than non-remitters (P < 0.0001) (**Table 1**).

**Table 1:** Comparison of endpoint clinical characteristics between remitters and non-remitters.

	Remitters (N = 227)	Non-remitters (N = 74)	Remitters and non-remitters (N = 301)	P
**Endpoint characteristics**				
PANSS-total score	35.9(5.8)	48.2(13.1)	38.9(9.8)	0.000
PANSS-positive score	7.4(1.1)	10.0(4.8)	8.0(2.8)	0.000
PANSS-negative score	9.5(3.0)	15.3(5.8)	10.9(4.6)	0.000
PANSS-general psychopathological score	19.0(3.0)	23.0(6.0)	20.0(4.3)	0.000
PSP	80.0(9.3)	68.3(12.4)	77.2(11.3)	0.000

PANSS, positive and negative syndrome scale. Theoretical scores range from 30 to 210 (total score), from 7 to 49 (positive scale), from 7 to 49 (negative scale), and from 16 to 112 (general psychopathology scale). Higher scores indicate more severe psychopathology. PSP, personal and social performance scale. Higher scores indicate better function.

### Demographic and Clinical Characteristics of the Sample

In the 301 patients who completed the first year of antipsychotic treatment, there was no difference between remitters and non-remitters in terms of baseline demographic and clinical characteristics, namely, psychiatric family history, illness duration, acute onset (less than 3 months from symptom onset to full disease manifestation), early onset (onset before 18 years) and concomitant antidepressants or mood stabilizers. Totally, 27 subjects accepted antidepressants, with fluoxetine equivalent dose (25.7 ± 8.9)mg (25, 26). Non-remitters were associated with more medication switch and higher daily antipsychotic dosage (in olanzapine equivalent) (27) during 1-year treatment, which indicated more treatment effort (**Table 2**).

**Table 2:** Baseline demographic characteristics and clinical characteristics of the population.

	Remitters N = 227	Non-remitters N = 74	Remitters and Non-remitters N = 301	p
Age (years)	24.8 (7.0)	24.9 (7.9)	24.8 (7.2)	0.527
Gender (Male)	126 (56.3%)	45 (60.8%)	171 (57.4%)	0.492
Marital status (Single)	184 (82.1%)	61 (82.4%)	245 (82.2%)	0.955
Education (years)	12.8 (2.8)	12.5 (2.8)	12.8 (2.8)	0.431
Positive psychiatric family history	17 (7.8)	10 (14.3)	27 (9.4)	0.105
Age of onset	24.2 (7.0)	24.4 (8.0)	24.2 (7.3)	0.638
Illness duration (months)	10.1 (10.3)	12.2 (11.2)	10.6 (10.6)	0.157
Acute onset	55 (29.6%)	15 (24.6%)	70 (28.3%)	0.454
Early onset	21 (22.6%)	12 (33.3%)	33 (25.6%)	0.209
Primary medication				0.556
Risperidone	86 (78.9%)	23 (21.1%)	109 (36.2%)	
Olanzapine	61 (72.6%)	23 (27.4%)	84 (27.9%)	
Aripiprazole	80 (74.1%)	28 (25.9%)	108 (35.9%)	
Daily dose (in olanzapine equivalent)	14.0 (4.2)	16.2 (3.5)	14.5 (4.1)	0.000
Medication switch	63 (27.8%)	32 (43.2%)	95 (31.6%)	0.013
Concomitant treatments				
Mood stabilizers	0	0	0	N/A
Antidepressants	19 (8.4%)	8 (10.8%)	27 (9.0%)	0.523

### Comparison Between Remitters and Non-Remitters

#### Univariate Analysis of the Baseline Characteristics

At baseline, the PANSS negative score was lower in remitters than in non-remitters; however, there were no differences in the PANSS total, positive and general psychopathological scores; PSP score; and cognitive performance (all P > 0.05) (**Table 3**). Of the seven items on the PANSS negative scale, the N2 “emotional withdrawal”, N4 “passive/apathetic”, N5 “abstract thinking” and N7 “stereotyped thinking” scores were lower in remitters (P < 0.01, P < 0.05, P < 0.01 and P < 0.05, respectively) than in non-remitters, but there were no differences in the N1 “blunted affect”, N3 “poor rapport” and N6 “lack of spontaneity” scores.

**Table 3:** Comparison of baseline characteristics between remitters and non-remitters.

	Remitters (N = 227)	Non-remitters (N = 74)	Remitters and non-remitters (N = 301)	P
**Baseline characteristics**				
PANSS-total score	85.7 (13.7)	88.3 (15.4)	86.3 (14.2)	0.161
PANSS-positive score	23.4 (5.2)	23.4 (5.3)	23.4 (5.2)	0.880
PANSS-negative score	20.3 (7.2)	22.9 (5.3)	23.4 (5.2)	0.007
PANSS-general psychopathological score	42.0 (7.6)	42.0 (7.6)	42.0 (7.6)	0.741
PSP	42.2 (12.3)	41.5 (17.0)	42.0 (13.6)	0.252
Verbal learning	36.9 (11.7)	39.0 (10.8)	37.4 (11.5)	0.233
Visual learning	38.1 (11.9)	37.3 (10.1)	37.9 (11.5)	0.657
Working memory and attention	36.6 (9.9)	35.4 (10.2)	36.3 (9.9)	0.418
Information processing speed	36.3 (8.3)	34.6 (9.4)	35.9 (8.6)	0.179
Executive function	33.1 (10.7)	32.2 (9.6)	32.9 (10.4)	0.573
Fine motor	27.2 (10.6)	26.3 (10.9)	27.0 (10.7)	0.577

All the abbreviations are explained in the first footnote to **Table 1**.

#### Multivariate Analysis of the Baseline Characteristics

In the multivariate analysis including the PANSS negative scale score (P <0.05, OR = 0.947[0.906, 0.990]), N2 “emotional withdrawal” (P <0.05, OR = 0.789[0.626, 0.995]), N4 (P <0.05, OR = 0.799[0.646, 0.990]), N5 (P <0.05, OR = 0.761[0.602, 0.961]), and N7 “stereotyped thinking” (P <0.05, OR = 0.745[0.588, 0.943]) scores, respectively with the 6 cognitive scores, their significance persisted.

In logistic regression analysis including N2 “emotional withdrawal”, N4 “passive/apathetic”, N5 “abstract thinking” and N7 “stereotyped thinking” as independent variables, N5 “abstract thinking” and N7 “stereotyped thinking” remained significant but N2”emotional withdrawal” and N4 “passive/apathetic” did not. Considering the potential overlap of N2 “emotional withdrawal” and N4 “passive/apathetic”, we conducted two additional logistic regression analyses including N2 “emotional withdrawal”, N5 “abstract thinking” and N7 “stereotyped thinking” and N4 “passive/apathetic”, N5 “abstract thinking” and N7 “stereotyped thinking” respectively, and the significance of N5 “abstract thinking” and N7 “stereotyped thinking” persisted.

## Discussion

Nearly 3/4 FES patients, who completed the 1-year routine antipsychotic treatment, achieved relatively stable clinical recovery and functional recovery. Patients with fewer negative symptoms had a better chance of PR. One higher score on item N2 “emotional withdrawal”, N4 “passive/apathetic”, N5 “abstract thinking” or N7 “stereotyped thinking” could reduce the possibility of PR by nearly 1/5 to 1/4.

### FES PRR Within 1 Year of Treatment

Gabeal et al. obtained an average PRR of 42.3% (2–3 years after the acute phase) based on the results from more than 10 FES studies conducted from 2006 to 2011 (5). A recent meta-analysis showed an FES symptomatic remission rate as high as 56.0% (16). All the studies mentioned above included the dropouts in the denominators when calculating the PRR. The PRR in this study was 39.9% (227/569) if the dropouts were included in the analysis, which is consistent with previous studies. This result suggests that more than 1/3 of FES patients achieved PR within the 1-year follow-up. However, including the dropouts in the denominator when calculating the PRR might lead to confusion. On the one hand, the dropouts may achieve PR after dropping out of the study, which might lead to underestimation of the rate. On the other hand, the rate could be greatly influenced by the dropout rate; thus, the PRR with the dropouts included might reflect a social or behavioral dimension rather than the outcome of antipsychotic treatment, although Lally et al. reported that remission in FEP was not associated with dropout rates (16).

When only patients who completed the 1-year follow-up were included in the denominators in RCTs, Boter et al. reported a PRR of 68.7% in the EUFEST study, and Crespo-Facorro et al. reported a PRR of 35.2% (9). Another RCT of 111 patients in Germany showed that the PRR was 58.6% (65/111); however, some patients also received psychological education or CBT for 8 weeks (5). The PRR in our study was much higher, which suggested that a relatively flexible regimen was needed to maximize the possibility of PR in FES.

One naturalistic follow-up study of 104 FEP patients in Hong Kong showed that the PRR was 59.6% (28), but this study did not define the antipsychotic treatment patients had received prior to recruitment. Our study recruited only FES patients with no or minimal antipsychotic exposure. Being previously untreated was a predictor of remission in general schizophrenia (11). The difference in the PRR between our study and the study by Chang et al. suggested that being previously untreated might also be a predictor of PRR in the FES population. Thus, most FES patients, who completed the 1-year antipsychotic treatment, achieved relatively stable symptomatic and functional outcomes.

### Predictors of PR in FES

This study indicates that PR in FES patients who completed the 1-year antipsychotic treatment was not associated with gender, age, onset age (5, 8, 16), medication (9), or education level (14), which was consistent with previous studies.

#### Cognitive Impairment and PR

Cognitive impairment is considered a diathesis factor in schizophrenia. However, Boter et al. failed to demonstrate that cognitive impairment predicted PR in the EUFEST study (8). Ventura et al. also showed that cognitive impairment, including in vocabulary, comprehension and visual information processing speed, was not associated with the PRR in the FEP population (29). Although Simonsen et al. suggested that baseline information processing speed—but not verbal memory, verbal fluency, working memory and interference control—was better in remitters than in non-remitters, the significance disappeared in a multivariate analysis (14). This study confirmed that cognitive impairment was not associated with PR within the first year of antipsychotic treatment.

#### Baseline Symptoms and PR

Notably, negative symptoms were the only independent predictor of PR within the first year of antipsychotic treatment, consistent with several previous studies (28). Baseline symptoms (especially negative symptoms) predicted remission in FES patients in a systemic review (4), although Ceskova et al. followed 93 male FES patients for 1 year and found no difference in the baseline PANSS total score or subscale scores between remitters and non-remitters (30). A more recent meta-analysis of FEP suggested that baseline psychotic symptoms were not associated with remission; however, it did not explore the potential effects of subgroup symptoms (16).

We found that negative symptoms rather than overall symptoms might predict PR and further revealed that “emotional withdrawal”, “passive/apathetic”, “abstractive thinking” and “stereotyped thinking” but not “blunted affect”, “poor rapport” and “lack of spontaneity and flow of conversation” predicted PR. This result suggested that only some negative symptoms are associated with PR.

“Emotional withdrawal” and “passive/apathetic” both refer to others’ observations and are used to assess volition impairment in patients with schizophrenia. There is abundant evidence demonstrating daily activity reduction in schizophrenia; however, the potential predictive power of these symptoms had not been explored previously.

It is interesting that the two items (“abstractive thinking” and “stereotyped thinking”) describing thinking impairment and not the items describing volition impairment were shown to independently predict PR. In other words, volition symptoms were mediated by thinking symptoms in their prediction of PR. P5 “abstractive thinking” refers to the ability to comprehend and abstract. N7 “stereotyped thinking” refers to the flexibility of thinking, that is, the ability to shift between two perspectives (or two beliefs about the world) according to the demands of the social situation. The Word Fluency Test in the cognitive assessment battery was used to assess the richness of thinking; however, performance on this test did not differ between remitters and non-remitters in a *post hoc* analysis. Thus, impairment in the ability to comprehend and abstract and in thinking flexibility rather than other thinking impairments (e.g., richness) might predict a lower likelihood of PR. Abstract thinking impairment is an established deficit in schizophrenia (31), and impairment in cognitive flexibility has been found in FES patients (32). This subgroup of negative symptoms has a high degree of overlap with cognitive symptoms. In fact, abstract thinking and stereotyped thinking are items of the cognitive rather than the negative component of the PANSS (33). It has been demonstrated that disorganization (with difficulty in abstract thinking as one of the three items) played a key (direct and indirect) role in real-life functioning in patients with schizophrenia (34). Comparelli et al., further demonstrated that difficulty in abstract thinking was correlated with social cognition and did not differ between early schizophrenia and late schizophrenia (35). However, there has been little research exploring the significance of these impairments in the prognosis of schizophrenia, though there has been some research exploring the underlying neural basis (32, 36). Our findings call for further studies with stricter designs to examine the predictive power of negative symptoms for PR, especially thinking symptoms and volition impairment rather than affective symptoms. Additionally, FES patients usually display mild or no negative symptoms, unlike chronic patients; indeed, in this study, the symptom severity as measured by the items on the negative subscale was generally “very mild” or “mild”. This might also explain why there was no difference in the perseveration error in the Wisconsin Card Sorting Test (WCST) or the completion time in the Color Trail II test between remitters and non-remitters in the *post hoc* analysis. However, these two variables reflect thinking flexibility in a rather limited and inflexible way, while the PANSS item N7 “stereotyped thinking” assesses comprehensive judgment of general information and thus better reflects mild impairment. Therefore, we speculate that even mild negative thinking or volition symptoms at baseline might predict a lower likelihood of PR in FES. More studies are needed to confirm this hypothesis.

Clinically, our findings suggested that FES patients with even mild difficulty in understanding others’ words or shifting their perspectives according to the social situation or who exhibit a mild reduction in daily activities might be less likely to remit during the first year of antipsychotic treatment, despite the severity of their other symptoms, including positive symptoms and negative affective symptoms.

The study had several limitations. First, the follow-up period was 1 year, so the results cannot be generalized to a longer period. However, Ceskova et al. found that in male patients with FES, the remission rates after 1, 4 and 7 years of treatment were comparable, and patients who did not remit within the first year of treatment did not remit after 4 or 7 years of treatment either (37). It is possible that remission within a 1-year treatment period could be used to predict subsequent remission. Second, we focused on patients who completed the whole year of antipsychotic treatment; thus, we failed to provide information about remission in FES patients who discontinued their antipsychotic treatment, that is nearly half of the whole group. Kurihara et al. (17), who followed schizophrenia patients for 17 years, revealed that all remitters had ceased taking antipsychotics, which suggested that patients who discontinued their antipsychotics could also remit. The remission rate and its predictors in these patients need to be explored *via* studies with special designs and large samples. Third, patients who did not continue the follow-up may have received treatment from other psychiatric hospitals, the remission rate and its predictors in this group of patients were beyond the study. Fourth, we did not employ the MATRICS Consensus Cognitive Battery (MCCB) in cognitive assessment which is the most widely used and standardized neuropsychological test battery for assessing cognition in schizophrenia worldwide and was in the process of development when this trial was conducted. Social cognition included in the MCCB was not assessed in this study. Fifth, this study included FES patients with an illness duration not exceeding three years; thus, whether patients with an illness duration longer than 3 years are less likely to remit could not be determined by this study.

## Conclusion

The present study showed that over 3/4 FES patients achieved PR within 1 year of antipsychotic treatment. Patients with no impairment in their comprehension ability, thinking flexibility and daily activities were more likely to achieve PR, indicating that thinking and volition negative symptoms define the subset of FES patients who are less likely to achieve ideal symptom outcomes in 1 year of antipsychotic treatment. Thus, assessing these symptoms in detail is necessary in clinical practice. Optimization of the current pharmacological and non-pharmacological strategies for treating this group of patients is warranted.

## Data Availability Statement

The datasets generated for this study are available on request to the corresponding author.

## Ethics Statement

The studies involving human participants were reviewed and approved by Peking University Sixth Hospital Ethics Committee. The patients/participants provided their written informed consent to participate in this study.

## Author Contributions

XY, YY, FY, ZL, CW, HD, and JZ designed the study. XY obtained funding and supervised the study. XZ and ZC analyzed the data. XY, ZC, and XZ interpreted the data and drafted the report. ZC, XH, LY, FY, ZL, CW, HD, JZ, and YY participated in the collection of data. All authors contributed to the article and approved the submitted version.

## Funding

This study was supported by National Key Project of Scientific and Technical Supporting Programs funded by Ministry of Science & Technology of China (No. 2007BAI17B04) and the Key Program of Beijing Science and Technology Commission (D171100007017002). CNFEST received study drugs from Eli Lily, Janssen & Janssen and Kanghong Pharmaceutical Co. Ltd. The funding agency of the study as well as the pharmaceutical companies providing the study medications in kind had no role in study design, data collection, data analysis, data interpretation, writing of the report, or decision where to publish the results.

## Conflict of Interest

XY has been an advisor or in speaker’s bureau to or has received honoraria from: Eli Lilly China, Xian Janssen, Lundbeck, Pfizer China, Eisai China, Novartis China and Sanofi China.

The remaining authors declare that the research was conducted in the absence of any commercial or financial relationships that could be construed as a potential conflict of interest.

## References

[1] Menezes NM Malla AM Norman RM Archie S Roy P Zipursky RB A multi-site Canadian perspective: examining the functional outcome from first-episode psychosis. Acta Psychiatr Scand (2009) 120(2):138–46. 10.1111/j.1600-0447.2009.01346.x 19207130

[2] Zhu Y Li C Huhn M Rothe P Krause M Bighelli I How well do patients with a first episode of schizophrenia respond to antipsychotics: A systematic review and meta-analysis. Eur Neuropsychopharmacol (2017) 27(9):835–44. 10.1016/j.euroneuro.2017.06.011 28669774

[3] Leucht S Heres S Kissling W Davis JM Evidence-based pharmacotherapy of schizophrenia. Int J Neuropsychopharmacol (2011) 14(2):269–84. 10.1017/S1461145710001380 21208500

[4] AlAqeel B Margolese HC Remission in schizophrenia: critical and systematic review. Harv Rev Psychiatry (2012) 20(6):281–97. 10.3109/10673229.2012.747804 23216066

[5] Gaebel W Riesbeck M Wolwer W Klimke A Eickhoff M von Wilmsdorff M Rates and predictors of remission in first-episode schizophrenia within 1 year of antipsychotic maintenance treatment. Results of a randomized controlled trial within the German Research Network on Schizophrenia. Schizophr Res (2014) 152(2-3):478–86. 10.1016/j.schres.2013.04.012 23643327

[6] Andreasen NC Carpenter WT Kane JM Lasser RA Marder SR Weinberger DR Remission in schizophrenia: proposed criteria and rationale for consensus. Am J Psychiatry (2005) 162(3):441–9. 10.1176/appi.ajp.162.3.441 15741458

[7] Jager M Messer T Laux G Pfeiffer H Naber D Schmidt LG Standardized remission criteria in schizophrenia: descriptive validity and comparability with previously used outcome measures. Pharmacopsychiatry (2008) 41(5):190–5. 10.1055/s-2008-1078745 18763221

[8] Boter H Peuskens J Libiger J Fleischhacker WW Davidson M Galderisi S Effectiveness of antipsychotics in first-episode schizophrenia and schizophreniform disorder on response and remission: an open randomized clinical trial (EUFEST). Schizophr Res (2009) 115(2-3):97–103. 10.1016/j.schres.2009.09.019 19819114

[9] Crespo-Facorro B Perez-Iglesias R Mata I Caseiro O Martinez-Garcia O Pardo G Relapse prevention and remission attainment in first-episode non-affective psychosis. A randomized, controlled 1-year follow-up comparison of haloperidol, risperidone and olanzapine. J Psychiatr Res (2011) 45(6):763–9. 10.1016/j.jpsychires.2010.11.002 21106207

[10] Perez-Iglesias R Crespo-Facorro B Martinez-Garcia O Ramirez-Bonilla ML Alvarez-Jimenez M Pelayo-Teran JM Weight gain induced by haloperidol, risperidone and olanzapine after 1 year: findings of a randomized clinical trial in a drug-naive population. Schizophr Res (2008) 99(1-3):13–22. 10.1016/j.schres.2007.10.022 18053689

[11] Gasquet I Haro JM Tcherny-Lessenot S Chartier F Lepine JP Remission in the outpatient care of schizophrenia: 3-year results from the Schizophrenia Outpatients Health Outcomes (SOHO) study in France. Eur Psychiatry (2008) 23(7):491–6. 10.1016/j.eurpsy.2008.03.012 18573640

[12] Haro JM Novick D Bertsch J Karagianis J Dossenbach M Jones PB Cross-national clinical and functional remission rates: Worldwide Schizophrenia Outpatient Health Outcomes (W-SOHO) study. Br J Psychiatry (2011) 199(3):194–201. 10.1192/bjp.bp.110.082065 21881098

[13] Kelly DL Weiner E Ball MP McMahon RP Carpenter WT Buchanan RW Remission in schizophrenia: the relationship to baseline symptoms and changes in symptom domains during a one-year study. J Psychopharmacol (2009) 23(4):436–41. 10.1177/0269881108093883 PMC3718069 18583442

[14] Simonsen C Faerden A Romm KL Berg AO Bjella T Sundet K Early clinical recovery in first-episode psychosis: Symptomatic remission and its correlates at 1-year follow-up. Psychiatry Res (2017) 254:118–25. 10.1016/j.psychres.2017.04.050 28460281

[15] Ucok A Serbest S Kandemir PE Remission after first-episode schizophrenia: results of a long-term follow-up. Psychiatry Res (2011) 189(1):33–7. 10.1016/j.psychres.2010.11.013 21196051

[16] Lally J Ajnakina O Stubbs B Cullinane M Murphy KC Gaughran F Remission and recovery from first-episode psychosis in adults: systematic review and meta-analysis of long-term outcome studies. Br J Psychiatry (2017) 211(6):350–8. 10.1192/bjp.bp.117.201475 28982659

[17] Kurihara T Kato M Reverger R Tirta IG Seventeen-year clinical outcome of schizophrenia in Bali. Eur Psychiatry (2011) 26(5):333–8. 10.1016/j.eurpsy.2011.04.003 21696927

[18] Han X Yuan YB Yu X Zhao JP Wang CY Lu Z The Chinese First-Episode Schizophrenia Trial: background and study design. East Asian Arch Psychiatry (2014) 24(4):169–73. 25482837

[19] Cheng Z Yuan YB Han X Yang L Cai S Yang F An open-label randomised comparison of aripiprazole, olanzapine and risperidone for the acute treatment of first-episode schizophrenia: Eight-week outcomes. J Psychopharmacol (2019) 33(10):1227–36. 10.1177/0269881119872193 31487208

[20] Cheng Z Yuan Y Han X Yang L Zeng X Yang F Rates and predictors of one-year antipsychotic treatment discontinuation in first-episode schizophrenia: Results from an open-label, randomized, “real world” clinical trial. Psychiatry Res (2019) 273:631–40. 10.1016/j.psychres.2019.01.068 30735922

[21] Kay SR Fiszbein A Opler LA The positive and negative syndrome scale (PANSS) for schizophrenia. Schizophr Bull (1987) 13(2):261–76. 10.1093/schbul/13.2.261 3616518

[22] Tianmei S Liang S Yun’ai S Chenghua T Jun Y Jia C The Chinese version of the Personal and Social Performance Scale (PSP): validity and reliability. Psychiatry Res (2011) 185(1-2):275–9. 10.1016/j.psychres.2010.05.001 20542575

[23] Nuechterlein KH Green MF Kern RS Baade LE Barch DM Cohen JD The MATRICS Consensus Cognitive Battery, part 1: test selection, reliability, and validity[J]. Am J Psychiatry (2008) 165(2):203–13. 10.1176/appi.ajp.2007.07010042 18172019

[24] Shi C Kang L Yao SQ Ma Y Li T Liang Y The MATRICS Consensus Cognitive Battery (MCCB): Co-norming and standardization in China[J]. Schizophr Res (2015) 169:109–15. 10.1016/j.schres.2015.09.003 PMC4916953 26441005

[25] Bollini P Pampallona S Tibaldi G Kupelnick B Munizza C Effectiveness of antidepressants. Meta-analysis of dose-effect relationships in randomised clinical trials. Br J Psychiatry (1999) 174:297–303. 10.1192/bjp.174.4.297 10533547

[26] Furukawa TA Cipriani A Cowen PJ Leucht S Egger M Salanti G Optimal dose of selective serotonin reuptake inhibitors, venlafaxine, and mirtazapine in major depression: a systematic review and dose-response meta-analysis. Lancet Psychiatry (2019) 6(7):601–9. 10.1016/S2215-0366(19)30217-2 PMC6586944 31178367

[27] Gardner DM Murphy AL O’Donnell H Centorrino F Baldessarini RJ International consensus study of antipsychotic dosing. Am J Psychiatry (2010) 167(6):686–93. 10.1176/appi.ajp.2009.09060802 20360319

[28] Chang WC Chan TC Chen ES Hui CL Wong GH Chan SK The concurrent and predictive validity of symptomatic remission criteria in first-episode schizophrenia. Schizophr Res (2013) 143(1):107–15. 10.1016/j.schres.2012.10.016 23151398

[29] Ventura J Subotnik KL Guzik LH Hellemann GS Gitlin MJ Wood RC Remission and recovery during the first outpatient year of the early course of schizophrenia. Schizophr Res (2011) 132(1):18–23. 10.1016/j.schres.2011.06.025 21764563 PMC3172347

[30] Ceskova E Radovan P Tomas K Hana K One-year follow-up of patients with first-episode schizophrenia (comparison between remitters and non-remitters). Neuropsychiatr Dis Treat (2007) 3(1):153–60. 10.2147/nedt.2007.3.1.153 PMC2654532 19300545

[31] Harrow M Adler D Hanf E Abstract and concrete thinking in schizophrenia during the prechronic phases. Arch Gen Psychiatry (1974) 31(1):27–33. 10.1001/archpsyc.1974.01760130013002 4835984

[32] Pardo BM Garolera M Ariza M Pareto D Salamero M Valles V Improvement of cognitive flexibility and cingulate blood flow correlates after atypical antipsychotic treatment in drug-naive patients with first-episode schizophrenia. Psychiatry Res (2011) 194(3):205–11. 10.1016/j.pscychresns.2011.06.001 22044531

[33] Si T Yang JZ Shu L Wang XL Kong QM Zhou M The reliability, validity of PANSS and its implication. Chin Ment Health J (2004) 18(1):45–7. 10.3321/j.issn:1000-6729.2004.01.016

[34] Galderisi S Rossi A Rocca P Bertolino A Mucci A Bucci P Italian Network For Research on, Psychoses. The influence of illness-related variables, personal resources and context-related factors on real-life functioning of people with schizophrenia. World Psychiatry (2014) 13(3):275–87. 10.1002/wps.20167 PMC4219069 25273301

[35] Comparelli A Corigliano V Forcina F Bargagna P Montalbani B Falcone G The Complex Relationship Among Formal Thought Disorders, Neurocognition, and Functioning in Nonacutely Ill Schizophrenia Patients. J Nerv Ment Dis (2020) 208(1):48–55. 10.1097/NMD.0000000000001087 31738225

[36] Oh J Chun JW Joon JOH Kim E Park HJ Lee B The neural basis of a deficit in abstract thinking in patients with schizophrenia. Psychiatry Res (2015) 234(1):66–73. 10.1016/j.pscychresns.2015.08.007 26329118

[37] Ceskova E Prikryl R Kasparek T Outcome in males with first-episode schizophrenia: 7-year follow-up. World J Biol Psychiatry (2011) 12(1):66–72. 10.3109/15622975.2010.518625 21087079

